# Tumor RNA transfected DCs derived from iPS cells elicit cytotoxicity against cancer cells induced from colorectal cancer patients in vitro

**DOI:** 10.1038/s41598-022-07305-1

**Published:** 2022-02-28

**Authors:** Shimpei Maruoka, Toshiyasu Ojima, Hiromitsu Iwamoto, Junya Kitadani, Hirotaka Tabata, Shinta Tominaga, Masahiro Katsuda, Keiji Hayata, Akihiro Takeuchi, Hiroki Yamaue

**Affiliations:** Second Department of Surgery, School of Medicine, Wakayama Medical University, 811-1, Kimiidera, Wakayama 641-8510 Japan

**Keywords:** Applied immunology, Gene therapy, Stem-cell biotechnology, Cancer, Gastroenterology, Oncology

## Abstract

Significant efficacy of induced pluripotent stem cells (iPSCs) in generating DCs for cancer vaccine therapy was suggested in our previous studies. In clinical application of DC vaccine therapy, however, few DC vaccine systems have shown strong clinical response. To enhance immunogenicity in the DC vaccine, we transfected patient-derived iPSDCs with in vitro transcriptional RNA (ivtRNA), which was obtained from tumors of three patients with colorectal cancer. We investigated iPSDCs-ivtRNA which were induced by transfecting ivtRNA obtained from tumors of three colorectal cancer patients, and examined its antitumor effect. Moreover, we analyzed neoantigens expressed in colorectal cancer cells and examined whether iPSDCs-ivtRNA induced cytotoxic T lymphocytes (CTLs) against the predicted neoantigens. CTLs activated by iPSDCs-ivtRNA exhibited cytotoxic activity against the tumor spheroids in all three patients with colorectal cancer. Whole-exome sequencing revealed 1251 nonsynonymous mutations and 2155 neoantigens (IC_50_ < 500 nM) were predicted. For IFN-γ ELISPOT assay, these candidate neoantigens were further prioritised and 12 candidates were synthesized. IFN-γ ELISPOT assay revealed that the CTLs induced by iPSDCs-ivtRNA responded to one of the candidate neoantigens. In vitro CTLs obtained by transfecting tumor-derived RNA into iPSDCs derived from three patients with colorectal cancer showed potent tumor-specific killing effect.

## Introduction

Cancer immunotherapy has entered a new era, and clinical applications with immune checkpoint inhibitors have greatly improved cancer treatment. Dendritic cells (DCs) are the most potent antigen-presenting cells, playing a significant role in initiating adaptive immune responses. Focusing upon these immune functions, DC-based cancer vaccine therapy has been developed as a strategy to enhance a patient’s immune system to eliminate cancer. In clinical application of DC vaccine therapy, however, DCs generated from patients with a variety of malignancies are impaired in terms of maturation and antigen-presenting ability^1,2^. The current DC vaccine therapy also requires a large amount of DCs derived from a patient’s peripheral blood monocytes, and frequent aphereses are a burden to the patients. Development of new methods to generate large amounts of DCs without patient-derived apheresis is urgently desired.

Since the discovery of induced pluripotent stem cells (iPSCs) technology in 2006, it has been possible to induce DCs differentiation from iPSCs. Several studies have subsequently sought to find various methods for deriving DCs from iPSCs (iPSDCs) in mouse and human studies^3–7^. Our previous studies have suggested the significant efficacy of iPSCs in generating DCs for cancer vaccine therapy^8,9^. Genetically-modified iPSDCs expressing tumor-associated antigen (TAA) was equivalent to that of bone marrow-derived DCs (BMDCs) in our mouse model in terms of functional and antigen-presenting capacity^8^. Genetically modified iPSDCs expressing carcinoembryonic antigen (CEA) that were induced by transducing CEA cDNA into iPSDCs were also shown to exhibit CEA-specific cytotoxicity in our mouse and human models^9^.

In clinical application of DC vaccine therapy, however, few DC vaccine systems targeting TAA have shown strong clinical response. The antitumor responses of DC vaccines targeting a single TAA have been reported to vary depending upon intra-tumoral heterogeneity^10^. In addition, when targeting a small number of TAA in these vaccine systems, there are no strong antitumor effects that cause regression of solid tumors^11^. For sufficient immunogenicity of iPSDCs in DC vaccine therapy, we suggest that generation of iPSDCs loaded with multiple TAAs is necessary.

In this study, we use amplified mRNA from human colorectal cancer cells, synthesized them by in vitro transcription^12,13^, and transfected iPSDCs with in vitro transcriptional RNA (ivtRNA). We examine whether the ivtRNA-transfected iPSDCs induce tumor-specific cytotoxic T lymphocytes (CTLs) and whether these antitumor responses are directed against multiple TAAs. An advantage of using antigen in the form of RNA is that RNA can be amplified from few cells, so a sufficient or even potentially unlimited amount of antigen can be generated from a small amount of tumor tissue. Trying to escape immune recognition, cancer undergoes gene mutations as the disease progresses by eliminating tumor antigens. We therefore seek to confirm whether neoantigens express in colorectal cancer cells and to prove whether ivtRNA-transfected iPSDCs induce CTLs against the predicted neoantigens. We verify whether iPSDCs-ivtRNA have immunoresponses against neoantigens, irrespective of this immune escape mechanism of cancer.

## Results

### Generation of iPSDCs

**Figure 1:**
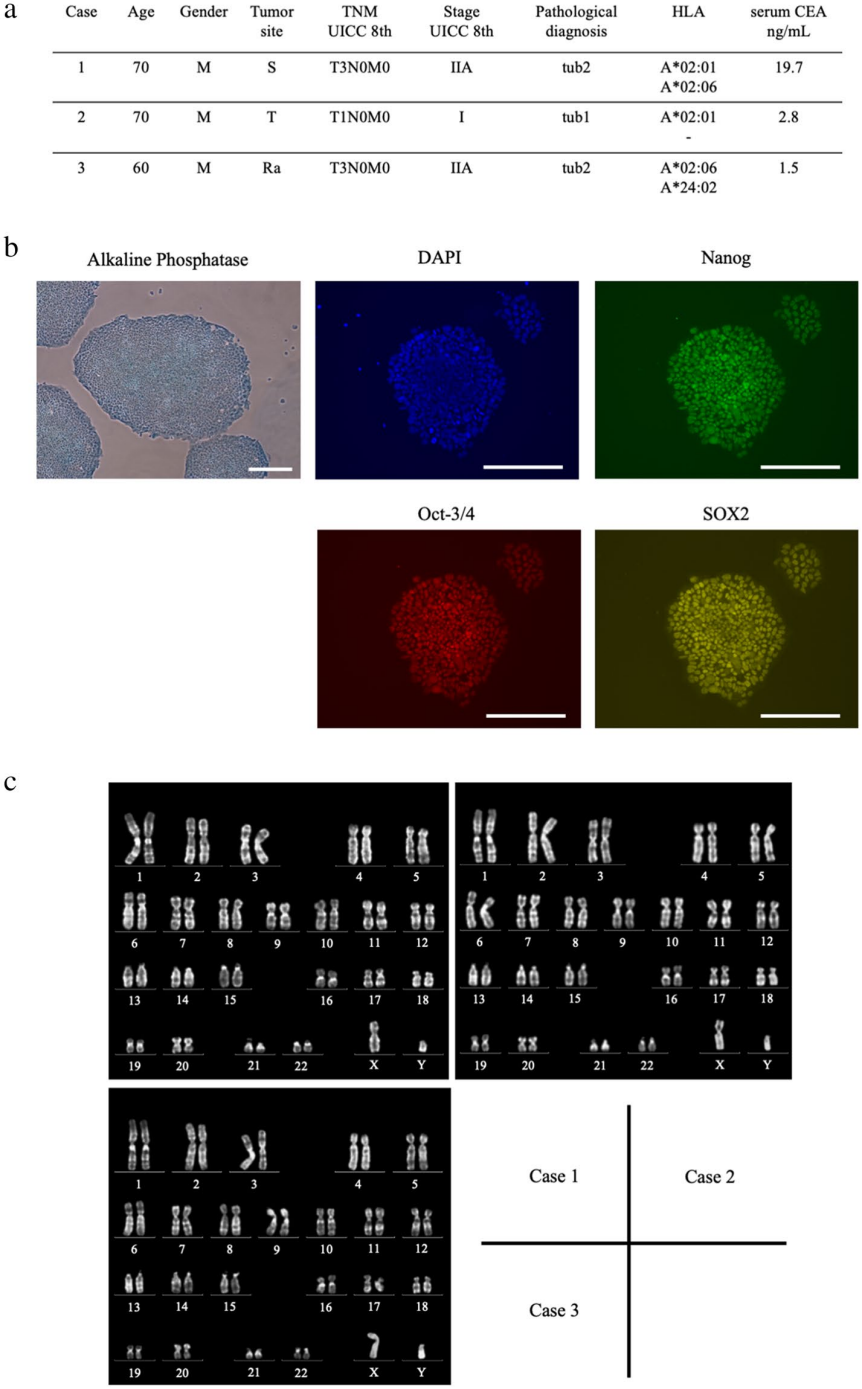

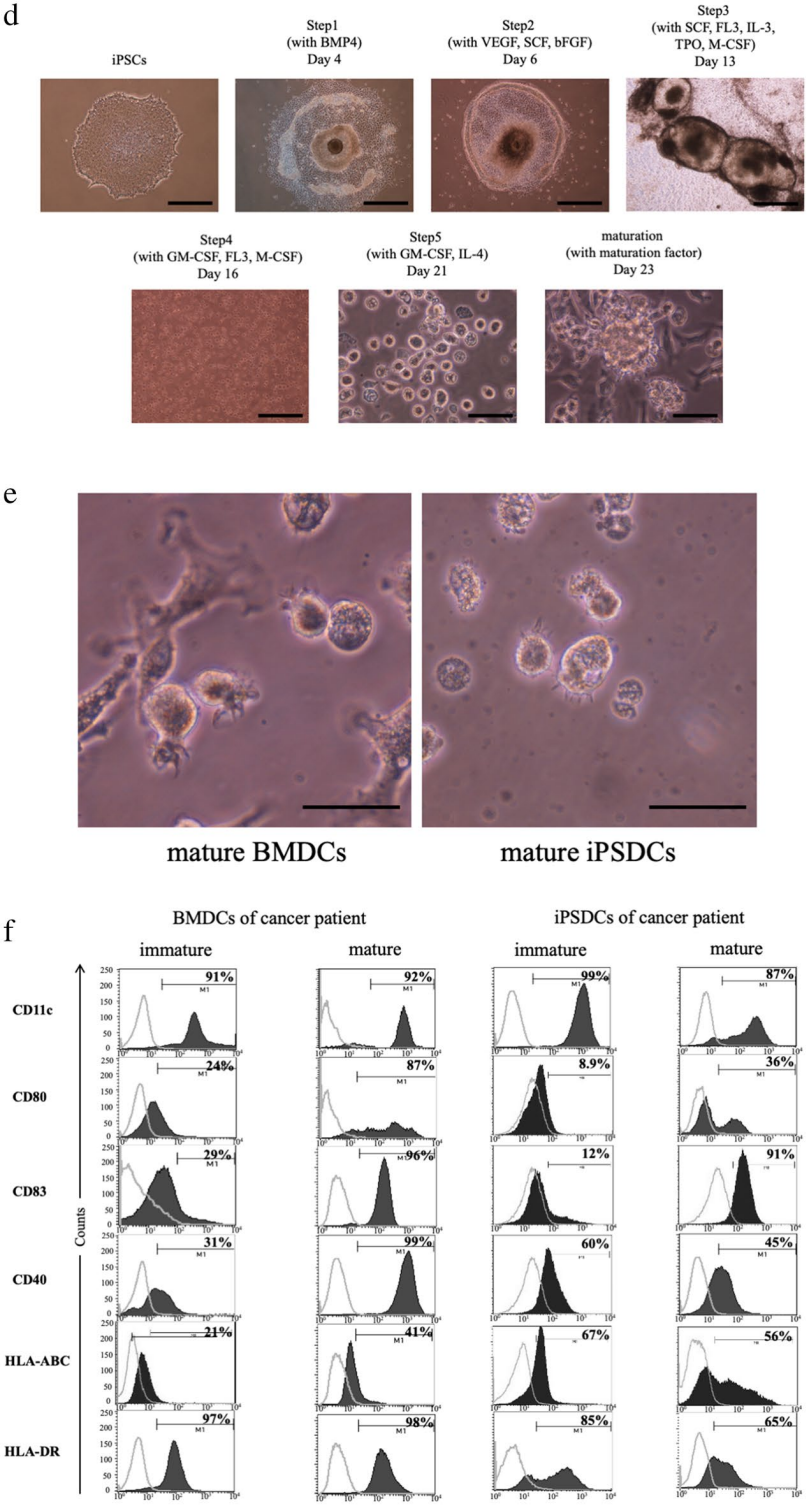

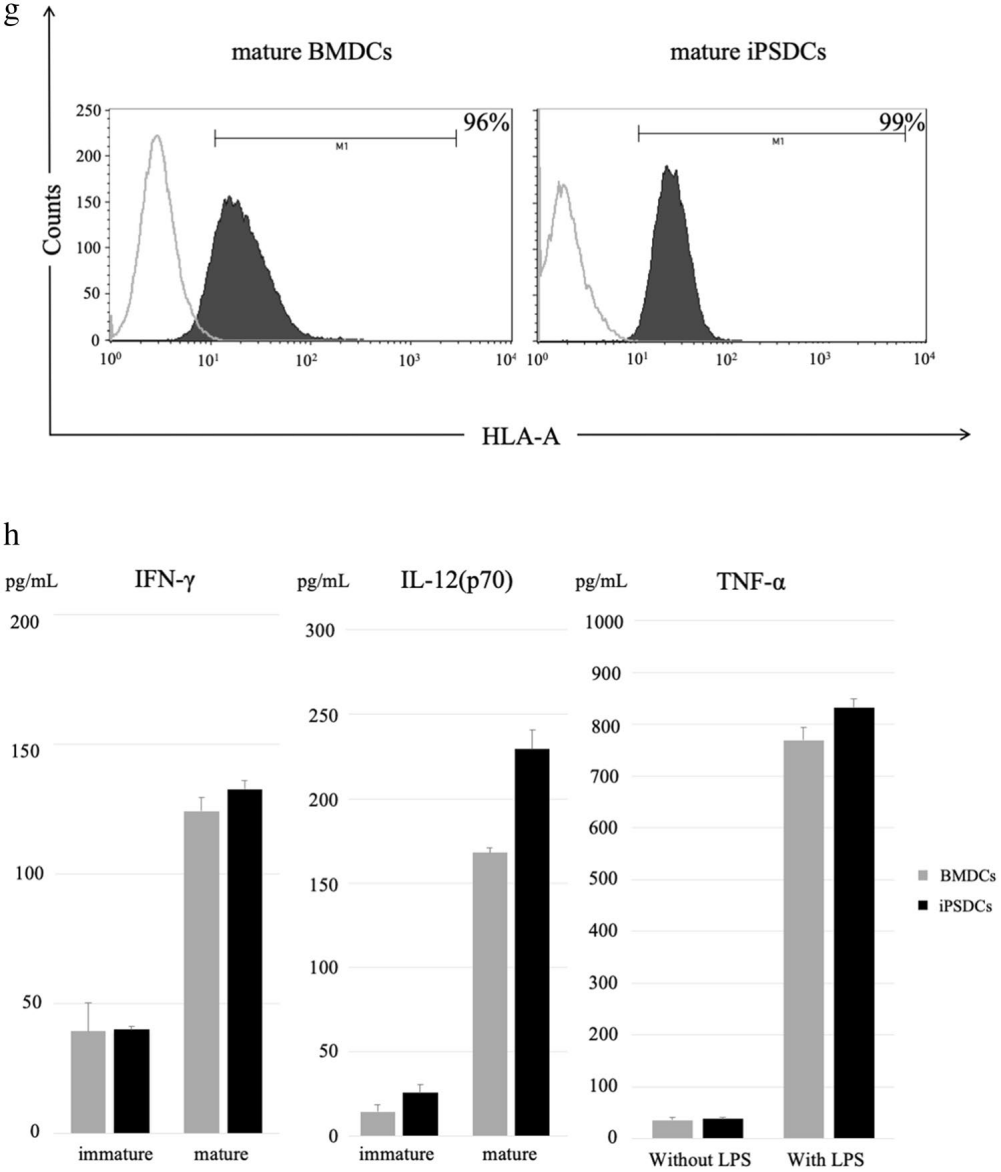
Generation of iPSDCs from patient-derived autologous iPSCs and their cell characterization in comparison to BMDCs. (**a**) List of colorectal cancer samples. The HLA-A allele was determined using an HLA-A High Resolution Typing System. Neoantigen analysis was performed in Case 3. Abbreviation Description S is sigmoid colon, T is transverse colon, Ra is upper rectum, tub1 is well-differentiated adenocarcinoma, and tub2 is moderately-differentiated adenocarcinoma. (**b**) Characterization of autologous iPSCs derived from a patient, Case 1. Alkaline phosphatase staining and fluorescent staining with undifferentiated markers showed pluripotency of iPSCs. Scale bars = 80 μm. (**c**) Karyotyping of iPSCs. No chromosomal abnormalities were found in these chromosomes in Case 1, 2, and 3. (**d**) The schematic diagram of differentiation protocol for iPSDCs. Scale bars = 80 μm (Before Day 16). Scale bars = 20 μm (After Day 21). (**e**) Morphology showing mature BMDCs (left) and mature iPSDCs (right) derived by Case 1. Scale bars = 20 μm. (**f**) Surface phenotypes of BMDCs (left) and iPSDCs (right) derived from the same cancer patient, Case 3. Black-filled histograms represent the staining results of specific antibodies. Gray lines highlight isotype-matched controls. (**g**) The expression of HLA-A on mature iPSDCs and BMDCs. Black-filled highlight the staining results of HLA-A. Gray lines histograms represent the isotype-matched controls. (**h**) The secretion of IFN-γ, IL-12(p70) and TNF-α from BMDCs and iPSDCs was examined by ELISA assay. Data represent the mean ± SD (three donors for each group).

Three patients with colorectal cancers were enrolled in this project, their characteristics are listed in Fig. 1a. Autologous iPSCs were generated from the PBMCs of the three patients using the Sendai virus vector^14,15^. It took 20–30 days to establish iPSCs. We then successfully induced differentiation of these iPSCs into DCs in 23 days. Alkaline phosphatase staining and fluorescent staining with undifferentiated markers showed the pluripotent status of iPSCs induced from these patients (Fig. 1b). Karyotyping was also performed on these iPSCs; no chromosomal abnormalities were identified (Fig. 1c). The iPSCs were maintained on tissue culture dishes coated with growth factor-reduced Matrigel in mTeSR1 serum-free medium.

In generating iPSDCs, we specifically used the following protocol, which consists of five sequential steps^9^. In step 1, primitive streak cells were induced from undifferentiated iPSCs. They were then differentiated into hemangioblastoma-like hematopoietic progenitors in step 2. After seven days, in step 3, dome-shaped structures were observed. After a further three days, in step 4, we verified that the majority of the floating cells were CD14 positive monocyte-like cells. Cells with protrusions appeared in step 5 of the immature DC stage, and then, 48 h after the addition of maturation cocktails (rhIL-6, rhTNFα, rhIL-1β, and Prostaglandin E2), the protrusion noticeably increased in the mature DC stage (Fig. 1d). Finally, it was confirmed that the resulting mature iPSDCs were morphologically similar to mature BMDCs freshly collected from the same patient (Fig. 1e). This protocol generated approximately 1 × 10^7^ iPSDCs per dish.

Flow cytometric analysis was performed to confirm cell surface markers of these iPSDCs. Mature iPSDCs generated from colorectal cancer patients as well as mature BMDCs collected from the same donor expressed high levels of CD11c. In the analysis of costimulatory molecules, CD83 had the similar expression rates (91% vs. 96%), but CD80 and CD40 had lower expression rates in iPSDCs. In the analysis of HLA, HLA-ABC and HLA-DR had lower expression rate in iPSDCs (Fig. 1f). The expression of HLA-A was comparable between iPSDCs and BMDCs (Fig. 1g). In a study of the secretion of INF-γ and IL12(p70) and TNF-α from DCs, the levels secreted from mature BMDCs and iPSDCs were higher than immature, but there were no differences in secretion level between the mature BMDCs and iPSDCs (Fig. 1h).

### Autologous CTOSs

**Figure 2:**
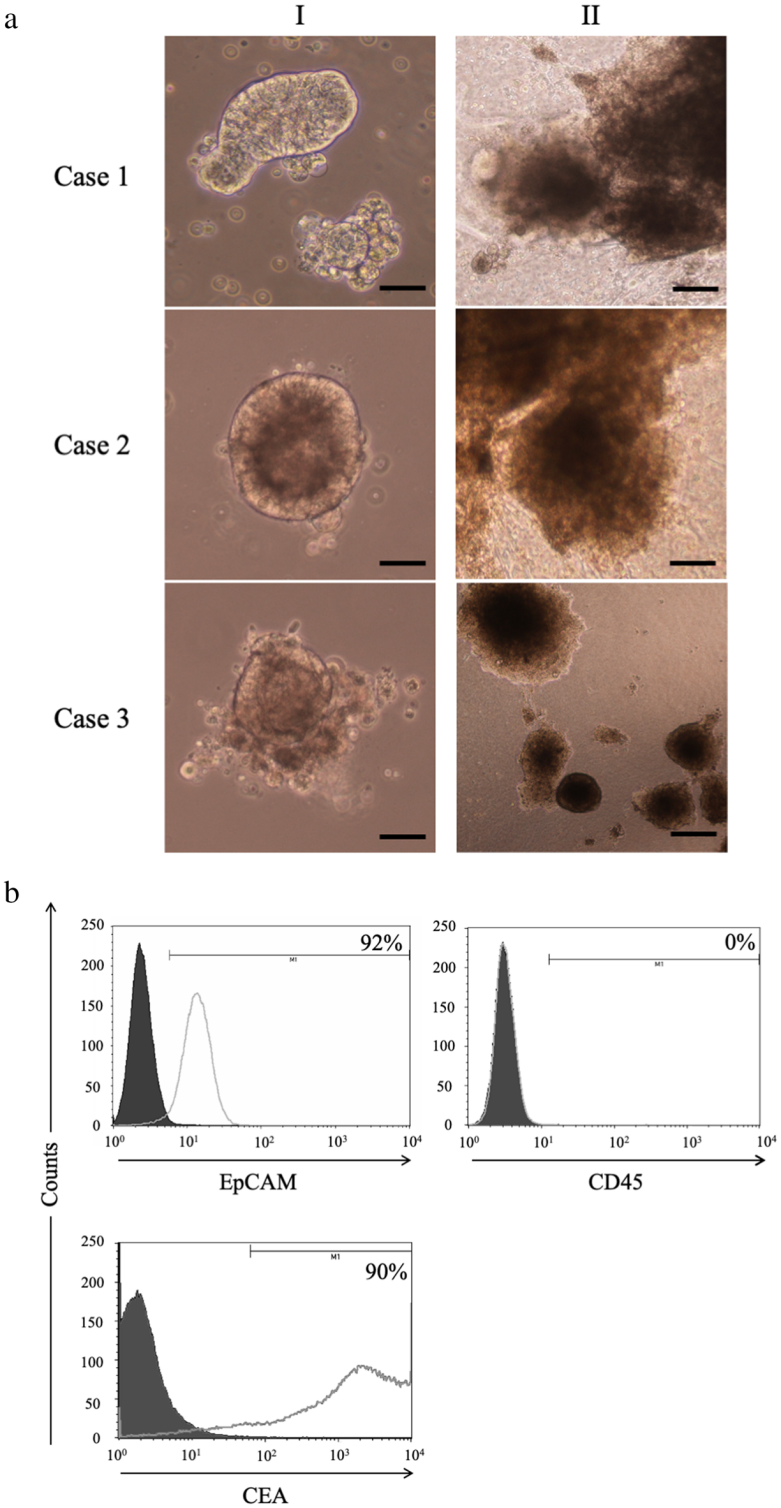
Establishment of Autologous CTOSs. (**a**) Formation of CTOSs from colorectal cancer tissues of three patients. Phase contrast images of organoid fraction: I, suspension culture; II, 3D culture. Scale bar = I, 100 μm; II, 40 μm. (**b**) Expression of EpCAM, CD45, and CEA in the CTOSs established from Case 1. Black-filled histograms represent the isotype-matched control. Gray lines highlight the staining results of EpCAM, CD45, and CEA.

Cancer tissue-originated spheroids (CTOSs) were established in all three patients with colorectal cancer (Fig. 2a). Suspension cultured CTOSs are shown in Figs. 2aI, 3d cultured CTOSs in Fig. 2aII. Expression of EpCAM, tumor cell marker, was observed in all patient-derived CTOSs and expression of CD45, blood cell marker, was not observed. The preoperative serum CEA value in Case 1 was high (19.7 ng/ ml), and the CTOSs established from Case 1 tumor expressed CEA at high frequency (Fig. 2b). In the other two cases, which had normal preoperative CEA, no expression of CEA was observed in these CTOSs.

**Figure 3:**
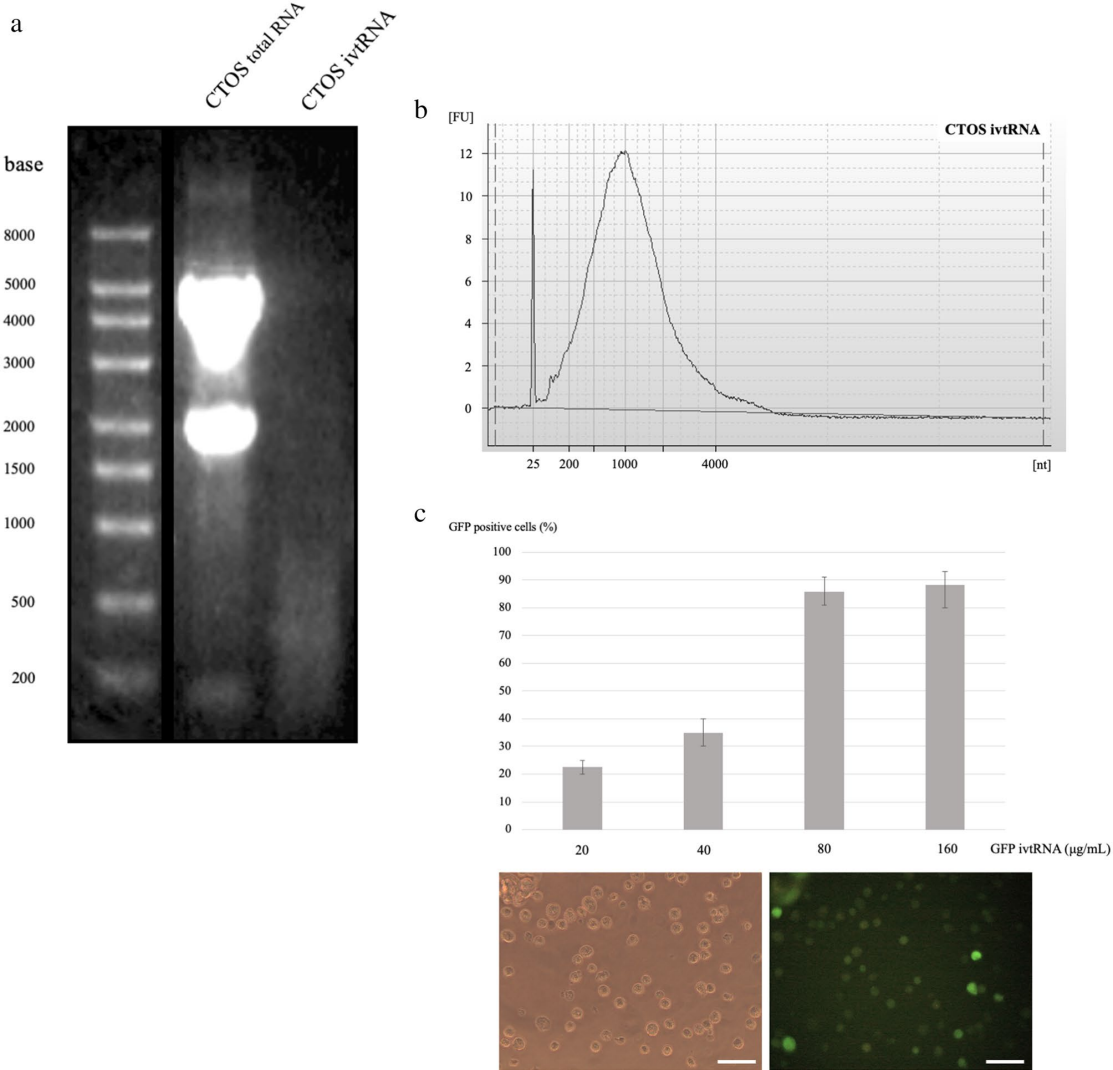
Analysis and functional evaluation of ivtRNA. (**a**) CTOSs total RNA and amplified CTOSs ivtRNA were subjected to agarose/formaldehyde gel electrophoresis and stained with ethidium bromide. Full-length blots/gels are presented in Supplementary information 5. (**b**) Microelectrophoresis curves from ivtRNA (Agilent 2100 bioanalyzer RNA6000 Pico Kit). (**c**) GFP expression rates at 24 h after electroporation of GFP ivtRNA. Experiments were performed in triplicate per condition. Phase contrast image (left) and fluorescent image (right) after 24 h with 80 μg/ ml GFP ivtRNA. Scale bars = 40 μm.

### Amplification of mRNA from CTOSs

Total RNA was isolated from CTOSs, and an aliquot of RNA was amplified. RNA was analyzed before and after amplification by agarose gel electrophoresis. Agarose gel electrophoresis under denaturing conditions display a smear present in ivtRNA (Fig. 3a). ivtRNA was normally distributed by bioanalyzer analysis (Fig. 3b).

### Electroporation of ivtRNA into mature iPSDCs

To determine transfection efficiency in mature iPSDCs and the relation between electroporated ivtRNA and expressed protein, we used different concentrations of ivtRNA coding for GFP. As shown in Fig. 3c, the amount of GFP positive cells was directly proportional to the ivtRNA concentration used during electroporation. Of the iPSDCs, 90% were GFP-positive at 80 μg/ ml ivtRNA concentration (Fig. 3c). For additional experiments, a concentration of 80 μg/ ml ivtRNA was chosen.

### Induction of tumor-specific CTLs and cytotoxicity in CTOSs model

**Figure 4:**
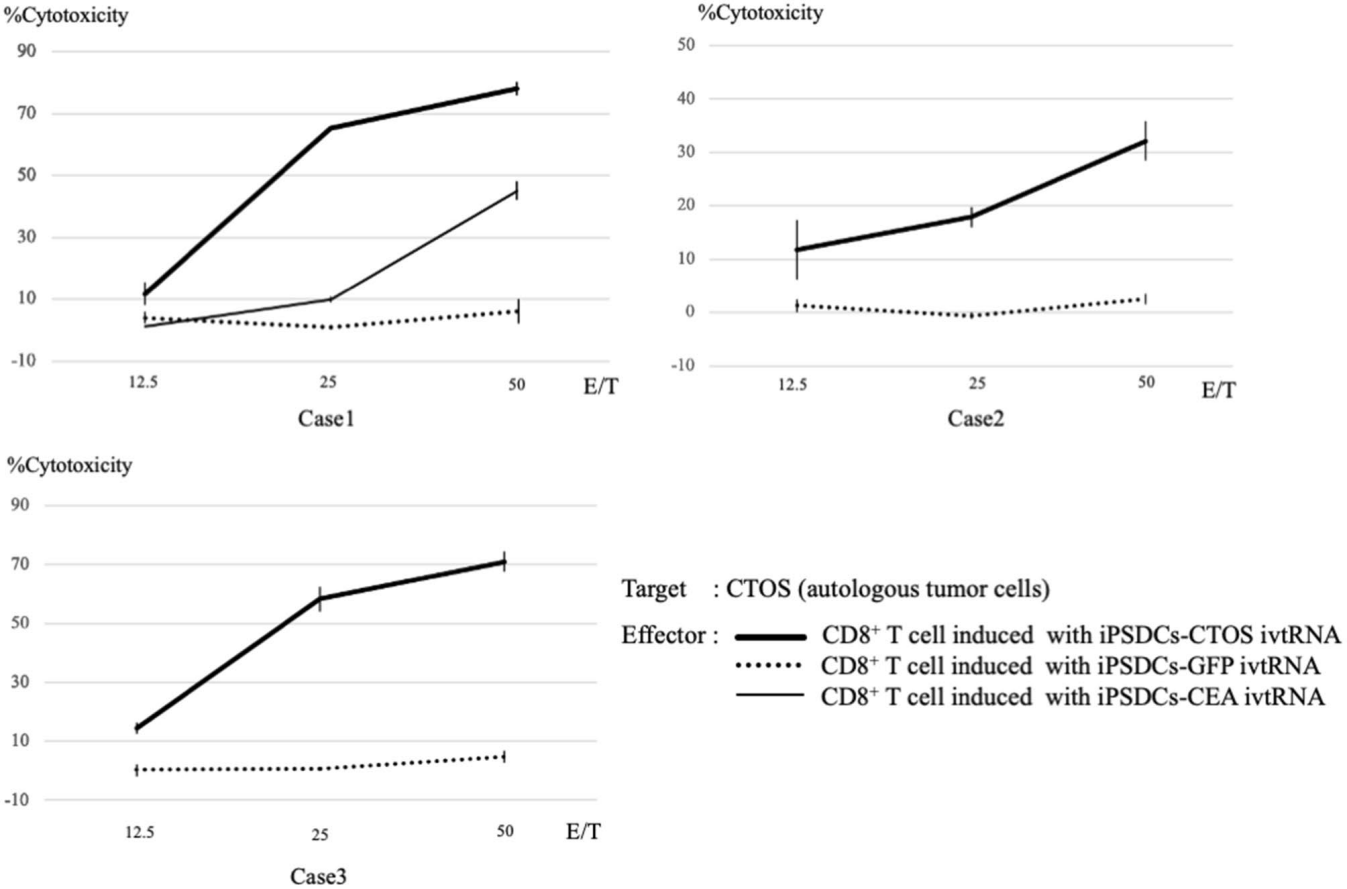
Cytotoxic activity of CTLs generated from iPSDCs-ivtRNA. CD8^+^ cytotoxic T cells were sorted using an autoMACS Pro Separator after three cycles of stimulation of autologous peripheral blood mononuclear cells, and their cytotoxic activity was tested using a 4-h _51_Cr release assay. The thick black line, dotted line, and thin black line represent the cytotoxic activity of CD8^+^ T cell induced with iPSDCs-CTOS ivtRNA, with iPSDCs-GFP ivtRNA, and with iPSDCs-CEA ivtRNA, respectively.

Inductions of CTOSs-specific CTLs were performed in each of the three donors. After three cycles of re-stimulation of PBMCs by iPSDCs-ivtRNA, CTLs were sorted and then assessed using a ^51^Cr-release assay. Three experiments from the three colorectal cancer donors showed similar results: the CTLs induced by iPSDCs-CTOS ivtRNA exhibited cytotoxic activity against CTOSs, but the CTLs induced by iPSDCs-GFP ivtRNA did not exhibit cytotoxic activity (Fig. 4). Our results showed that CTLs induced by iPSDCs-CTOS ivtRNA could recognize CTOSs. The cytotoxic activity of single antigen in Case 1 was also investigated, where CTOSs expressed CEA with high frequency. The CTLs induced with iPSDC-CEA ivtRNA showed cytotoxic activity against autologous CTOSs. The CTLs induced by iPSDCs-CTOS ivtRNA exhibited higher cytotoxic activity against CTOSs than the CTLs induced by iPSDCs-CEA ivtRNA (Fig. 4 in Case 1). These results suggest that the CTLs induced by iPSDCs-CTOS ivtRNA exhibit strong tumor-specific cytotoxic activity.

### Whole-exome sequencing and neoantigen prediction

Whole-exome sequencing was conducted to identify somatic mutations in CTOSs and normal cell DNA derived from Case 3 colorectal cancer tissues. Whole-exome sequencing first revealed 1251 nonsynonymous mutations (Supple. 1). Amino acid substitutions (AAS) corresponding to each of the nonsynonymous mutations were then translated to 8- to 11-mer amino acids and evaluated through the HLA class I peptide binding algorithm (NetMHC 3.4). After that, 2155 AAS-encoded neoantigens (IC_50_ < 500 nM) were predicted (Supple. 2). The transcriptional status of the AAS-encoded neoantigens was determined by RNA-seq. For experimental validation, predicted neoantigens were further prioritized based on the predicted HLA-A binding affinity and the difference in HLA-A affinity between AAS-peptide and WT-peptide and RNA expression rates (Supple. 3). Finally, 12 candidate neoantigens were synthesized and used in the following ELISpot assay (Supple. 4).

### T cell recognition of candidate neoantigens

**Figure 5:**
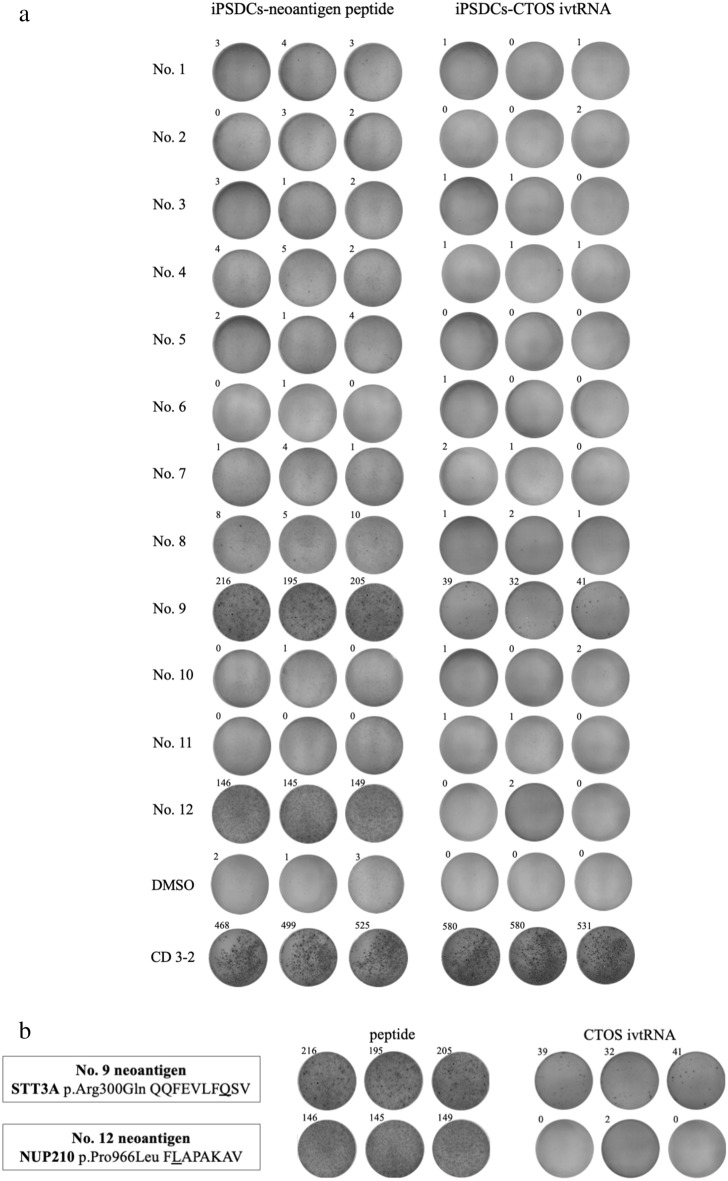
ELISpot assay of iPSDCs-neoantigen peptide and iPSDCs-CTOS ivtRNA. (**a**) IFN-γ secretion by CTLs induced by iPSDCs-neoantigen peptide and iPSDCs-CTOS ivtRNA against autologous dendritic cells co-cultured with neoantigen peptide. ELISpot experiments were performed in triplicate wells per condition. (**b**) Results of #9 and #12 candidate neoantigen peptide. The CTLs induced by iPSDCs-CTOS ivtRNA responded to #9 candidate neoantigen. Meanwhile, CTLs induced by iPSDCs-neoantigen peptide responded to #9 and #12 candidate neoantigens. The experiment was repeated twice with three wells, and the results were the same.

IFN-γ ELISpot assay was performed to determine whether CTLs induced by iPSDCs-CTOS ivtRNA recognize candidate neoantigen peptide. For positive control, we used mAb CD3-2. The CTLs induced by iPSDCs-CTOS ivtRNA responded to the #9 candidate neoantigen. Meanwhile, CTLs induced by iPSDCs-neoantigen peptide responded to the #9 and #12 candidate neoantigens (Fig. 5a,b). The experiment was repeated twice with three wells, and the results were the same. iPSDCs-CTOS ivtRNA induced neoantigen responses were therefore observed.

## Discussion

Despite its promise and a broad effort in the academia and biotech, DC vaccine therapy has not been satisfactorily successful. In this study, we integrated the two technological platforms, iPSDCs and ivtRNA isolated and amplified from patients with colorectal cancer.

Altogether, our studies clearly demonstrate that the high immunogenicity of iPSDCs-ivtRNA was generated by transfecting tumor RNA. By transfecting ivtRNA of CTOSs prepared from surgical specimens into iPSDCs generated from patients with colorectal cancer, we succeeded in inducing CTLs in vitro for the first time. In addition, these CTLs showed an immune response to a neoantigen.

This research exclusively used autologous, full HLA-matched cancer tissues. In examining the cytotoxic activity, we used CTOSs as the target cells because they are very similar to patient-derived cancer cells, reflecting their clinical heterogeneity^16^. Examination outcomes of cytotoxic activity in CTOSs can be reasonably assumed to reflect the results of practical clinical trials more accurately than the studies that use homogenous cultured cells.

In this study, the transfection efficiency of iPSDCs was examined by an electroporation method, thereby deciding the optimum concentration of ivtRNA at 80 μg/ ml. The electroporation parameters for this research were shown to be very effective in obtaining a high viability of iPSDCs and substantially preserving the cell surface markers level on these iPSDCs that pertains to antigen-presentation capacity^17^. Based on these electroporation parameters, iPSDCs-ivtRNA was generated from three cases of CTOSs derived from colorectal cancer and induced CTLs in vitro. These CTLs were then confirmed to have outstanding antitumor effects against the CTOSs derived from the patient tumor. In Case 1, where CTOSs expressed CEA, this study compared CTL antitumor immune response directed solely against CEA (iPSDCs-CEA ivtRNA) with that against multivalent antigens (iPSDCs-CTOS ivtRNA). There was higher cytotoxic activity in iPSDCs-CTOS ivtRNA. This research may be verified the high antitumor effects of iPSDCs-ivtRNA in comparison with the existing vaccines targeting a single TAA.

Neoantigens of Case 3 were also analyzed. His colorectal cancer contained tumor-specific frameshift mutation in MSH6 and as many as 1251 nonsynonymous mutations. MSH6 is one of the DNA mismatch-repair genes that play a significant role in repairing mis-incorporated bases and DNA damage, and it is reported that frameshift variants in MSH6 are observed in colorectal cancer at a particular frequency^18,19^.

Moreover, after selecting twelve candidate neoantigens, ELISpot assay was performed for these candidate neoantigens in Case 3. Selection criteria of candidate neoantigen in this study was basically established by referring to the existing reports^20–24^. However, the level of binding affinity of the predicted neoantigens to HLA molecules was made stricter. This is because Case 3 tumor cells have high mutation burden due to frameshift mutation in MSH6, and when the existing criteria are adapted, there are too many candidate neoantigens. We set the conditions for the level of binding affinity of the predicted neoantigens to HLA molecules more strictly. In previous reports^23,24^, the IC_50_ was generally less than 50 nM, but under our conditions it is less than 10 nM, and peptides that are predicted to bind more strongly to the HLA molecule are selected as candidate neoantigens. In the ELISpot assay of iPSDCs-neoantigen peptide, spot-formation was observed in the cell cultures, to which #9 and #12 predicted neoantigen peptides were added, respectively. Meanwhile, in an assay of iPSDCs-CTOS ivtRNA, spot-formation was observed with #9 predicted neoantigen. With regard to #9 predicted neoantigen, the number of spots found in the iPSDCs-neoantigen peptide were compared with that of iPSDCs-CTOS ivtRNA; a smaller number of spots were observed in iPSDCs-CTOS ivtRNA. This outcome can be interpreted as iPSDCs-neoantigen peptide, which was generated by being pulsed with peptide, was more capable of inducing antigen-specific T cells than iPSDCs-ivtRNA that was made by being transfected with mRNA by electroporation^25^. The actual vaccine effect is unknown, however, because there has been no comparison of the cytotoxic activity of CTLs obtained from antigen stimulation of iPSDCs-CTOS ivtRNA and iPSDCs-neoantigen peptide. This is an area for future study. On the other hand, spot formation was not observed in the iPSDCs-CTOS ivtRNA with #12 predicted neoantigen, perhaps because the quality control at a stage of ivtRNA generation had potential implications for this result, and perhaps because no NUP210 mutation was contained in CTOSs that was used for ivtRNA generation, because CTOS is a heterogeneous spheroid^26,27^. Further studies are thus required.

This research is the first of its kind using patients with cancer. Clinical application of DC vaccine therapy is hindered by the need for a large quantity of DCs generated from peripheral blood monocytes of the patient. The difficulty in obtaining a sufficient number of functional DCs is a well-known serious problem in DC based immunotherapy. By using the iPSDCs and ivtRNA as we are doing, production of a large amount of potent DC vaccines in a relatively short period of time is possible. In our differentiation method, approximately 1 × 10^7^ DCs can be prepared per dish, and the minimum time required to create iPSDCs-ivtRNA from a blood sample of a cancer bearing patient is about 45 days. Cancer undergoes gene mutations as the disease progresses while trying to escape immune recognition by eliminating tumor antigens^22^. Regardless of this immune escape mechanism of cancer, we demonstrated that iPSDCs-ivtRNA is expected to have antitumor effects, even against neoantigens. However, there are some limitations to this study. First, it is an in vitro study, so the possibility that our experiment might not precisely replicate an organism’s cellular conditions must be considered. We are currently planning to study an actual vaccine model of iPSDCs-ivtRNA in vivo. Regarding the maturation of iPSDCs, the expression of CD83 increased after stimulation with the maturation cocktail used in this experiment, and there was no difference between iPSDCs and BMBCs. However, the expression of other costimulatory molecules and HLA molecules in iPSDCs is lower than that of BMDCs, and further studies on the maturation method are required. In addition, the expression of the surface makers of iPSDCs in cancer-bearing patients is different among individuals, and it is necessary to improve the maturation method so that the expression is constant. The sample size was also limited, neoantigen analysis was just one case. The cancer cells of Case 3 had high mutation burden, which was advantageous for neoantigen analysis studies^28^. Currently, neoantigen analysis is being performed on another patient. Lastly, the outcome of the #12 neoantigen assay does not necessarily indicate whether ivtRNA fully reflected the transcriptome of the tumor. Instead, the possibility that iPSDCs-ivtRNA failed to present the neoantigen whose immune response was supposed to take place should be cautiously considered.

Finally, in vitro CTLs obtained by transfecting tumor-derived RNA into iPSDCs derived from three patients with colorectal cancer showed potent tumor-specific killing effect.

In the future, we are considering establishing iPSCs for each individual patient. This is because autologous iPSDCs are considered to be capable of presenting antigens on other HLA molecules such as HLA-B, C, and HLA-DR. It is very important to consider HLA-DR. Two recent clinical trials have shown promising results for a new personalized neoantigen-based vaccine in patients with glioblastoma. The majority of responses to this vaccination were induced by CD4 + T cells rather than CD8 + T cells, against predicted neoantigens^29,30^. We will continue to study other HLA loci in the future. Ultimately, our findings revealed that iPSDCs-ivtRNA paves the way for a promising DC vaccine therapy.

## Methods

### Generation of iPSDCs

Three patients with colorectal cancer were enrolled in this project, each providing signed informed consent. They all underwent surgery for colorectal cancer at Wakayama Medical University Hospital. This study protocol was approved by the Institutional Review Board of the Ethical Committee on Human Research at WMUH (approval no. 2657 UMIN000038017). All research has been performed in accordance with the Declaration of Helsinki. All three patients have given their consent for publication in an online open access publication. We collected 15 ml of blood and 1.2 × 10^7 peripheral blood mononuclear cells (PBMCs) from these three donors. iPSCs were derived from donor PBMCs using CytoTune-iPS 2.0 (ID Pharma) in accordance with the manufacturer’s instructions^14,15^. iPSCs colonies were formed in about 20 days. We confirmed the undifferentiated and pluripotent status of iPSCs by evaluating alkaline phosphatase staining and fluorescent staining with undifferentiated markers (R&D Systems) according to the manufacturer’s instructions. The differentiation protocol of the iPSDCs was modified from a previously established protocol^7,9^. In brief, in step 1, undifferentiated iPSCs were disseminated onto a 100-mm culture dish coated with growth factor-reduced Matrigel (CORNING) in mTeSR1-cGMP medium (STEMCELL Technologies) supplemented with 80 ng/mL rhBMP4 (R&D Systems). In step 2, on day 4, mTeSR1 medium was replaced with StemPro-34 serum-free medium (Gibco) containing 2 mM L-glutamine supplemented with 80 ng/mL rhVEGF (R&D Systems), 25 ng/mL basic FGF (FUJIFILM Wako), and 100 ng/mL rhSCF (PEPROTECH). In step 3, on day 6, the cytokines in StemPro-34 were changed to cytokines mixed with 50 ng/mL rhSCF, 50 ng/mL rhIL-3 (R&D Systems), 5 ng/mL rhTPO (PEPROTECH), 50 ng/mL rhM-CSF (PEPROTECH), and 50 ng/mL rhFlt-3 ligand (PEPROTECH). In step 4, on day 13, the cytokines in StemPro-34 were changed to cytokines mixed with 50 ng/mL rhM-CSF, 25 ng/mL rhGM-CSF (PEPROTECH), and 50 ng/mL rhFlt3 ligand. CD14 positive monocytic lineage cells were sorted using an autoMACS Pro Separator with CD14 MicroBeads, human (Miltenyi) on days 16 to 28. In step 5, 1.5 × 10^6 CD14 positive monocytic cells/well in six-well Costar Ultra-Low Attachment Surface plates (CORNING) were cultured in the StemPro-34 medium containing 25 ng/mL rhGM-CSF and 40 ng/mL rhIL-4 (R&D Systems) for 5 days for differentiation into immature iPSDCs. Immature iPSDCs were matured in the presence of 100 ng/mL rhIL-6 (R&D Systems), 10 ng/mL rhTNFα (R&D Systems), 10 ng/mL rhIL-1β (R&D Systems), and 1 μg/mL Prostaglandin E2 (Sigma Aldrich) to induce final maturation for 48 h.

### Tumor cell line preparation

For the primary culture of the cancer cells from patients with colorectal cancer, we established CTOSs, as described previously^26^. In brief, surgical specimens were minced into 2 mm cubes using scalpel blades and washed several times with Hank’s balanced salt solution (HBSS). Tumor fragments were digested with 0.26 U/mL Liberase DH solution (Roche Diagnostics) at 37 °C for 1 h 45 min, followed by adding Recombinant DNase I (Takara Bio) for 15 min. The partially digested tissue was filtered through a 500-μm mesh filter and a 250-μm mesh filter. The filtrate was filtered through a 100-μm strainer and a 40-μm strainer. The tumor tissue retained in the strainer was collected, washed twice with HBSS, and transferred to CTOS medium [18 mL DMEM/F-12 with GlutaMAX medium (Gibco) supplemented with 400 μL StemPro hESC SFM Supplement (Gibco), 8 ng/mL bFGF, 20 ng/mL EGF (FUJIFILM Wako), 0.1 mM 2-mercaptoethanol, 100 units/mL penicillin, 100 μg/mL streptomycin, and 25 μg/mL Amphotericin B]. CTOSs were cultured in suspension in a stem cell medium. After suspension culture, 3D culture was performed. For 3D culture, the CTOSs were embedded in Cellmatrix type I-A (Nitta Gelatin) droplets on untreated tissue culture dishes and overlaid with stem cell medium. The medium was changed two or three times a week. For further analysis or passage, CTOSs were released from the Cellmatrix after 1–2 weeks of cultivation by incubation with 0.2 mg/mL Collagenase Type IV (STEMCELL Technologies). For expansion, CTOSs were cut into cell clusters using 23-gauge needles, and the cell clusters were transferred to the fresh stem cell medium.

### Total RNA isolation and amplification

Total RNA was isolated from CTOSs using the RNeasy plus micro kit and QIAshredder (Qiagen). Isolated RNA was amplified and prepared for in vitro transcription, as described previously^12,13^. Briefly, CTOSs mRNA was reverse transcribed using SuperScript II reverse transcriptase (Thermo Fisher Scientific). The first-strand cDNA synthesis was performed with 10 pmol of a modified oligo-dT primer [5'-AAGCAGTGGTATCAACGCAGAGTACT(30)VN-3'], where V is G, A, or C and N is G, A, T, or C (Sigma Aldrich). To this, we added DTT, reaction buffer, dNTP mixture (Takara Bio), SUPERase In RNase Inhibitor (Thermo Fisher Scientific). The reaction was incubated at 42 °C for 30 min, and then at 10 pmol the T7 strand switch primer [5'-CTAATACGACTCACTATAGGGCGGG-3'] was added. The reaction was continued for 30 min and was stopped by placing it on ice. Second-strand cDNA synthesis was performed using an Advantage 2 PCR enzyme system (Takara Bio) with RNase H, from *E. Coli* (Thermo Fisher Scientific). The cDNA was amplified by placing 2 μl of the RT reaction into a 100 μl PCR reaction containing 20 pmol of the following primers: T7 PCR (5'- CCATCCTAATACGACTCACTATAGGGC-3') and 3' PCR (5'- AAGCAGTGGTATCAACGCAGAGT-3'). Cycling conditions were as follows: an initial 1 min denaturing step at 95 °C, followed by cycling at 95 °C for 30 s, 65 °C for 30 s, 68 °C for 6 min, and a final extension at 68 °C for 7 min; this was performed for 18 cycles. The amplified cDNA was purified with a MinElute PCR Purification Kit (Qiagen).

### In vitro transcription of amplified cDNA

In vitro transcription was performed using the T7 mMESSAGE mMACHINE Kit (Thermo Fisher Scientific)^12,13^. The reaction was carried out at 37 °C for 4 h, followed by the addition of TURBO DNase (Thermo Fisher Scientific) and incubation for 15 min. For the polyadenylation of in vitro transcriptional RNA, we used a Poly(A) Tailing Kit (Thermo Fisher Scientific). Amplified mRNA was purified with a RNeasy MinElute Cleanup Kit (Qiagen). RNA quality was verified by agarose gel electrophoresis and a Nanodrop 2000 (Thermo Fisher Scientific), and 2100 Bioanalyzer (Agilent Technologies), and then stored at − 80 °C.

### Preparation of CEA and GFP in vitro transcription RNA

For CEA ivtRNA, we purchased Human CEACAM5/CEA/CD66e Gene ORF cDNA clone expression plasmid (Sino Biological). Linearization with Xba I (NIPPON GENE), followed by in vitro transcription with the T7 mMESSAGE mMACHINE Kit, yielded a transcript that contains 2109 nucleotides corresponding to the coding region of CEA.

For green fluorescent protein (GFP) ivtRNA, we purchased GFP mRNA (OZ Biosciences). This product is polyadenylated RNA at the 3' end. It was therefore possible to prepare ivtRNA by the amplification and in vitro transcription protocol.

### Transfection of iPSDCs with ivtRNA

Mature iPSDCs were harvested and washed once with PBS (all at room temperature). The cells were resuspended in Opti-MEM without phenol red (Thermo Fisher Scientific) at a concentration of 5 × 10^6/ ml. RNA was transferred to a 4-mm cuvette (80 μg/ ml final concentration). A volume of 200 μl of cell suspension was added and incubated for 3 min before being pulsed in a Gene Pulser Xcell (Bio-Rad). Pulse conditions were square-wave pulse, 500 V, 0.5 ms^17^. Immediately after electroporation, the cells were transferred to Step 5 differentiation medium (the StemPro-34 medium containing 25 ng/ ml rhGM-CSF and 40 ng/ ml rhIL-4).

### Flow cytometric analysis

For BMDCs and iPSDCs staining, the following monoclonal antibodies were used: PE-conjugated anti-human CD11c, FITC-conjugated anti-human CD80, FITC-conjugated anti-human CD83, PE-conjugated anti-human CD40, PE-conjugated anti-human HLA-ABC, PE-conjugated anti-human HLA-DR (all from BD Biosciences), primary antibodies against HLA-A were Anti-HLA A antibody, and secondary antibodies were Goat Anti-Rabbit IgG H&L (FITC) (all from Abcam). For CTOSs staining, the following monoclonal antibodies were used: FITC anti-human EpCAM (Miltenyi), PE anti-human CD45 (BD Biosciences). Primary antibodies against CEA were anti-human CEA monoclonal antibody clone COL-1 (Leica Biosystems), and secondary antibodies were FITC-conjugated anti-mouse IgG polyclonal antibody (Agilent Technologies).

### Assays for cytokine secretion

The BMDCs and iPSDCs were adjusted to a concentration of 2.0 × 10^5 cells/well and cultured on a 48-well plate for 48 h in AIM-V Medium (Thermo Fisher Scientific) (1 ml/well). The supernatants were then harvested, and the human IFN-γ and human IL-12 (p70) and human TNF-α levels were measured using an INF-γ ELISA kit and an IL-12 (p70) ELISA kit and a human TNF-α kit, respectively (all from Thermo Fisher Scientific). In the TNF-α assay, 100 ng/ ml of LPS (Sigma Aldrich) was used to stimulate DCs.

### Induction of CD8^+^ cytotoxic T cells

For iPSDCs-ivtRNA, CD8^+^ cytotoxic T cells were induced from autologous PBMCs. On day 0, a total of 4 × 10^6 PBMCs and 2 × 10^5 iPSDCs-ivtRNA were mixed in AIM V Medium containing 10 ng/ ml rhIL-7 (CellGenix) and cultured in a 24-well plate at a total volume of 1 ml/ well. On day 2, AIM V Medium containing 20 U/ ml of rhIL-2 (PEPROTECH) was added at a total volume of 2 ml/ well. On days 7 and 14, the cultures were re-stimulated with iPSDCs-ivtRNA at a ratio of 20:1. After three cycles of re-stimulation, CD8^+^ cytotoxic T cells were sorted from the stimulated PBMCs on day 21 using an autoMACS Pro Separator and then used for the subsequent experiments^9^.

For iPSDCs-neoantigen peptide, iPSDCs were pulsed with 20 μg/ ml of the respective synthesized more than 95% purity neoantigen peptides (Hokkaido System Science) for 16 h at 37 °C, and treated with 30 μg/ ml of mitomycin C (Sigma Aldrich) at 37 °C for 30 min. Following washing out the residual peptides and mitomycin C, iPSDCs were cultured with autologous CD8^+^ T cells in 0.5 ml of CellGenix GMP DC Medium (CellGenix) with 5% Human serum AB male (ABS) (Biowest) supplemented with 30 ng/ ml rhIL-21 (CellGenix) on 48-well plate (each well contained 1.0 × 10^5 peptide pulsed iPSDCs, 5 × 10^8 CD8^+^ T cells) on day 1. Three days later (day 4), 5 ng/ ml rhIL-7 and 5 ng/ ml rhIL-15 (PEPROTECH) were added in the culture media^31–33^. On day 6, the cultures were transferred to a 12-well plate with DC Medium/5% ABS containing 5 ng/ ml IL-7 and 5 ng/ ml rhIL-15^34^. On day 8, cultures were supplemented with DC Medium/5% ABS containing 10 ng/ ml rhIL-7 and 10 ng/ ml rhIL-15. On day 11, neoantigen-specific T cells were assessed using an ELISpot assay.

### 4-h ^51^Cr release assay

The cytotoxic activity of CD8^+^ cytotoxic T cells was tested using a 4-h ^51^Cr release assay, as described previously^35,36^. CTOSs were used as target cells. Spontaneous release was determined by incubating target cells alone in CTOS medium. The total release was determined by incubating target cells with 150 μl of 1 M HCl (Sigma Aldrich). CD8^+^ cytotoxic T cells were incubated at different ratios with the ^51^Cr-labeled CTOSs (E:T ratios of 50:1, 25:1, 12.5:1). Percentage cytotoxicity was calculated according to the formula: % Lysis = (experimental cpm – spontaneous cpm/total cpm – spontaneous cpm) × 100. Duplicate measurements of three-step titrations of effector cells were used for all experiments.

### Whole-exome and transcriptome analysis

Case 3 underwent neoantigen analysis. Whole-exome and transcriptome were outsourced to Cancer Precision Medicine (CPM, https://www.cancerprecision.co.jp). Genomic DNAs and total RNAs were extracted from frozen tumors using the AllPrep DNA/RNA mini kit (Qiagen) according to the manufacturer’s instructions. Control genomic DNAs were extracted from peripheral blood samples using QIAamp DNA Blood Midi Kit (Qiagen). Whole-exome libraries were built up, as previously described^37^ and sequenced by 100-bp paired-end reads on HiSeq2500 Sequencer (Illumina). The obtained sequence data were analysed by CPM pipeline, as previously described^24^. Somatic variants (single nucleotide variations (SNVs) and indels) were called using the following parameters, (i) base quality of ≥ 15, (ii) sequence depth of ≥ 10%, (iii) variant depth of ≥ 2, (iv) variant frequency in tumor of ≥ 10%, (v) variant frequency in normal of < 2%, and (vi) Fisher *P* value of < 0.05^38^. SNVs and indels were annotated based on RefGene using ANNOVAR.

### Identification of potential neoantigens

Human Leukocyte Antigen (HLA) class I genotypes were determined by OptiType algorithm^39^ using whole-exome data of possible 8- to 11-mer peptides harboring each amino acid substitution to HLA class I molecules. They were then filtered out with the predicted binding affinity to HLA molecules lower than 500 nM, using NetMHCv3.4 software. In addition, we used the transcriptome data to further select non-synonymously mutant peptides with a defined level (at least 10 reads among ~ 20,000,000 sequence reads) of gene expressions in tumor cells. For experimental validation, candidates were further prioritized based on the following criteria: (1) high predicted affinity (< 10 nM) somatic single nucleotide variations, (2) affinity of mutant type peptides ≥ wild type peptides, and (3) high mutant RNA expression (≥ 100)^20–24^.

### IFN-γ ELISpot assay

ELISpot assay was performed using Human IFN-γ ELISpot^PRO^ kit (Mabtech) according to the manufacturer’s instructions. Briefly, plates were washed four times with PBS and blocked with CellGenix GMP DC Medium containing 10% of ABS at least 30 min before use. For co-culture, 1 × 10^4 responder CD8^+^ T cells for detection of CD8^+^ T-cell responses were co-cultured with 5 × 10^3 stimulator autologous BMDCs. Responder CD8^+^ T cells were educated by iPSDCs-neoantigen peptide or iPSDCs-CTOS ivtRNA. Autologous PBMCs were generated using the plastic adherence method, as described previously^34^ and then pulsed with the respective neoantigen peptides (20 μg/ ml). After that, they were directly added to the ELISpot wells and incubated with responder CD8^+^ T cells overnight in DC Medium containing 10% of ABS at 37 °C. The mAb CD3-2 was used as a positive control. After 24 h of co-culture, cells were removed from the plate, washed five times with PBS, and then diluted with anti-human IFN-γ mAb (7-B6-1-Biotin, Mabtech) 1:200 in filtered PBS containing 0.5% ABS. After rinsing, TMB substrate solution was used to develop the immunospots. Spots were captured and analyzed by an automated ELISpot reader, ImmunoSpot S4 Software package, Version 6.0.0.2 (Cellular Technology Limited).

## Supplementary Information

Supplementary Information 1.

Supplementary Information 2.

Supplementary Information 3.

Supplementary Information 4.

Supplementary Information 5.

Supplementary Information 6.

Supplementary Information 7.

Supplementary Information 8.

Supplementary Information 9.

Supplementary Information 10.

## Data Availability

The source data that supports the findings of this study are available from the corresponding author upon request.
