# Supplementary material for: Tumor RNA transfected DCs derived from iPS cells elicit cytotoxicity against cancer cells induced from colorectal cancer patients in vitro
Source: Sci Rep. 2022 Feb 28;12:3295. doi: 10.1038/s41598-022-07305-1 (PMC8885822; doi:10.1038/s41598-022-07305-1)
Supplement: Supplementary file 1 — Supplementary Information 1. [file 41598_2022_7305_MOESM1_ESM.docx]

Tumor RNA transfected DCs derived from iPS cells elicit cytotoxicity against cancer cells induced from colorectal cancer patients *in vitro*

Shimpei Maruoka^1^, Toshiyasu Ojima*^1^, Hiromitsu Iwamoto^1^, Junya Kitadani^1^, Hirotaka Tabata^1^, Shinta Tominaga^1^, Masahiro Katsuda^1^, Keiji Hayata^1^, Akihiro Takeuchi^1^,

Hiroki Yamaue^1^

^1^ Second Department of Surgery, School of Medicine, Wakayama Medical University, Wakayama, Japan.

*Correspondence and reprints:

Toshiyasu Ojima, M.D. Ph.D., Second Department of Surgery, School of Medicine, Wakayama Medical University 811-1, Kimiidera, Wakayama 641-8510, Japan.

Phone: 81-73-441-0613,

Fax: 81-73-446-6566,

E-mail:tojima@wakayama-med.ac.jp

**Supplementary information. 1**

**Mutations derived by comparing Whole-exome sequencing data in cancer cells and normal cells. “Unknown” cannot show the detailed effects of mutation because the region translated into the protein is unknown.**
