# Supplementary figures and images for: Tumor RNA transfected DCs derived from iPS cells elicit cytotoxicity against cancer cells induced from colorectal cancer patients in vitro
Source: Sci Rep. 2022 Feb 28;12:3295. doi: 10.1038/s41598-022-07305-1 (PMC8885822; doi:10.1038/s41598-022-07305-1)

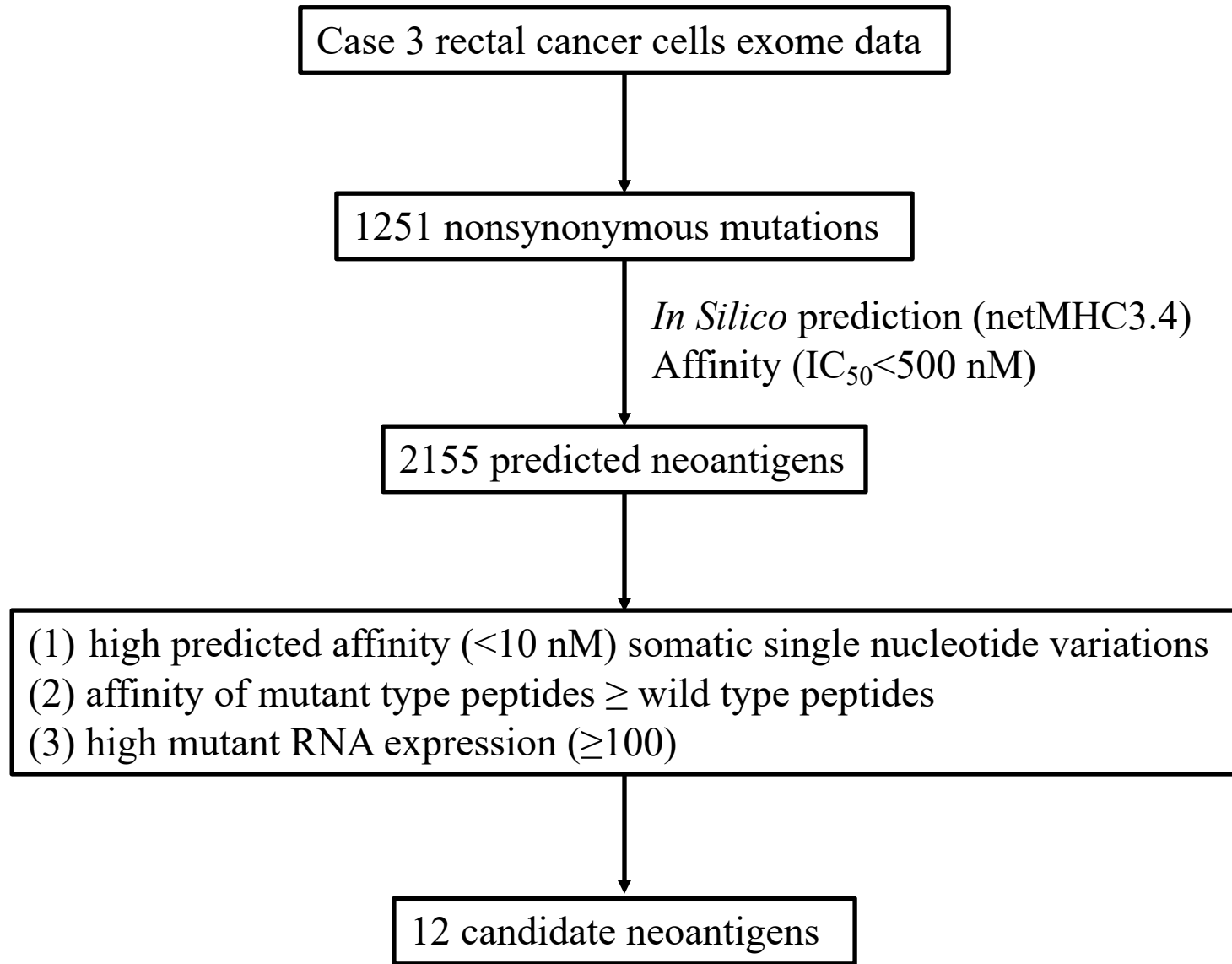

Supplement: Supplementary file 6 — Supplementary Information 6. [file 41598_2022_7305_MOESM6_ESM.pdf]

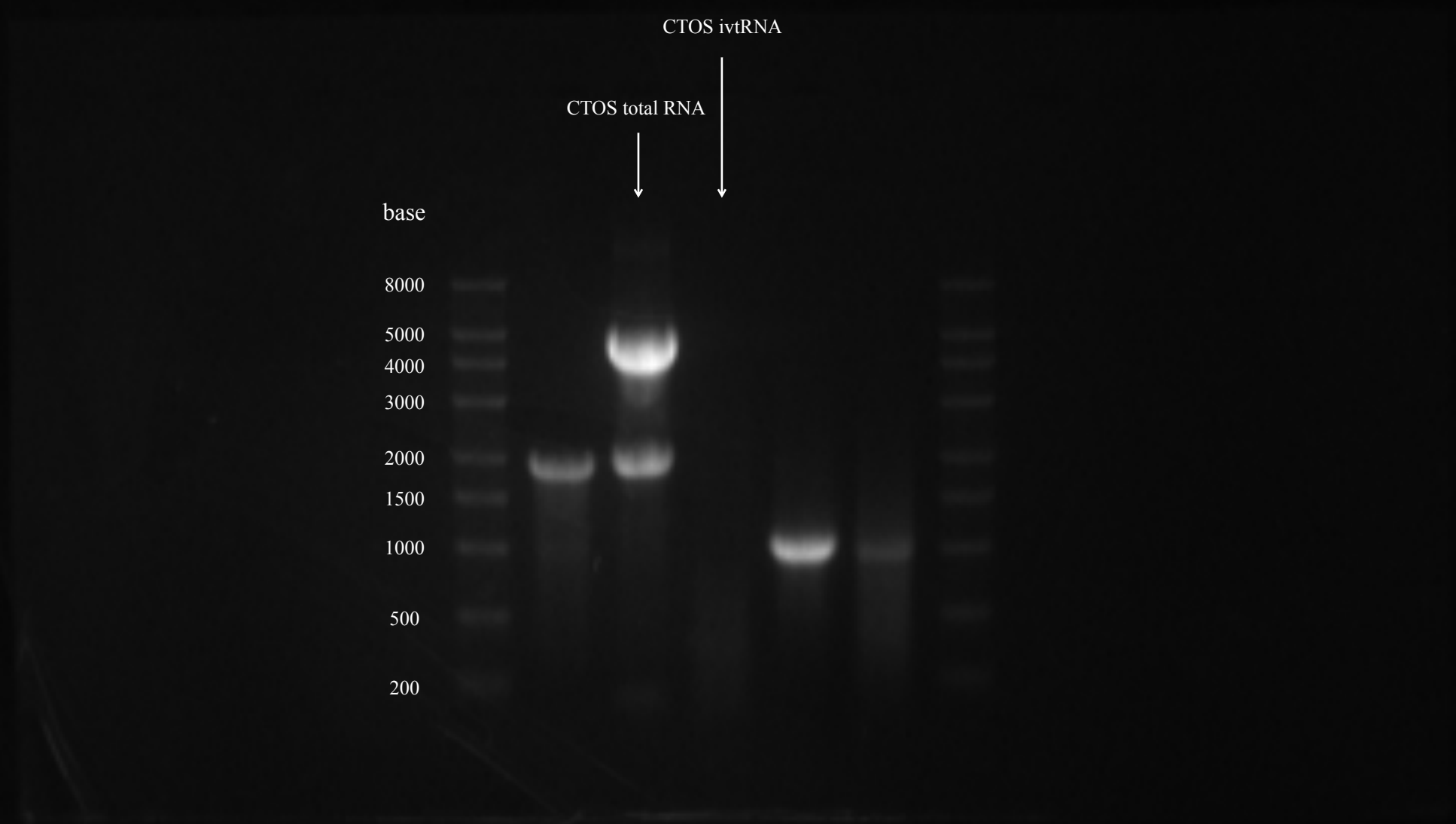

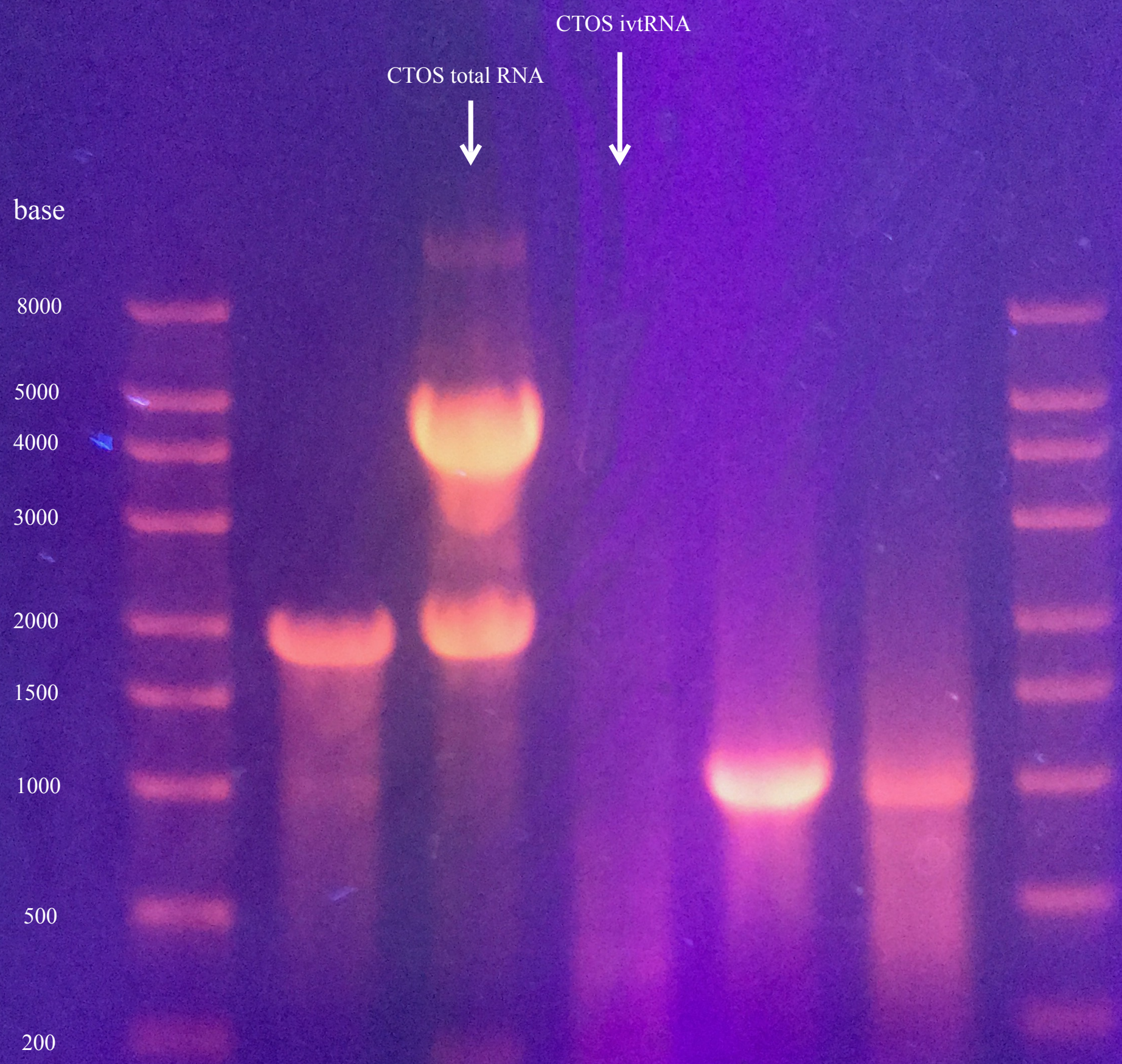

Supplement: Supplementary file 10 — Supplementary Information 10. [file 41598_2022_7305_MOESM10_ESM.pdf]
