# Supplementary material for: Tumor RNA transfected DCs derived from iPS cells elicit cytotoxicity against cancer cells induced from colorectal cancer patients in vitro
Source: Sci Rep. 2022 Feb 28;12:3295. doi: 10.1038/s41598-022-07305-1 (PMC8885822; doi:10.1038/s41598-022-07305-1)
Supplement: Supplementary file 8 [file 41598_2022_7305_MOESM2_ESM.docx]

nonsynonymous	1251
splicing	19
stopgain	70
stoploss	1
synonymous	575
unknown	14
frameshift deletion	63
frameshift insertion	24
nonframeshift deletion	1
splicing	3
stopgain	2
total	2023
