# Supplementary material for: Tumor RNA transfected DCs derived from iPS cells elicit cytotoxicity against cancer cells induced from colorectal cancer patients in vitro
Source: Sci Rep. 2022 Feb 28;12:3295. doi: 10.1038/s41598-022-07305-1 (PMC8885822; doi:10.1038/s41598-022-07305-1)
Supplement: Supplementary file 9 [file 41598_2022_7305_MOESM8_ESM.docx]

no.	gene	amino_acid	length	peptide_mut	affinity_mut(nM)	peptide_wt	affinity_wt(nM)	HLA	mutRNA_expression
1	NMT1	A283V	9	FQAVYTVGV	2	FQAVYTAGV	2	HLA-A02:06	139
2	PARP4	L785F	10	FTMSIEMPYV	3	LTMSIEMPYV	8	HLA-A02:06	277
3	SMAD2	T8M	9	SILPFMPPV	3	SILPFTPPV	3	HLA-A02:06	145
4	PWP1	R456Q	9	LQVWDISTV	4	LRVWDISTV	2001	HLA-A02:06	131
5	STT3A	R300Q	8	FQSVISLV	4	FRSVISLV	1911	HLA-A02:06	421
6	EFTUD2	T112M	8	MVYEMDFL	6	TVYEMDFL	24	HLA-A02:06	175
7	EFTUD2	T112M	9	MVYEMDFLA	6	TVYEMDFLA	24	HLA-A02:06	175
8	PLXNB2	V1284I	8	YTDRIFFL	6	YTDRVFFL	6	HLA-A02:06	151
9	STT3A	R300Q	10	QQFEVLFQSV	6	QQFEVLFRSV	8	HLA-A02:06	421
10	NUP210	P966L	9	FLAPAKAVV	7	FPAPAKAVV	5472	HLA-A02:06	180
11	IFRD2	R180C	9	FLLERCLTL	8	FLLERRLTL	16	HLA-A02:06	222
12	NUP210	P966L	8	FLAPAKAV	8	FPAPAKAV	3709	HLA-A02:06	180
